# Resistance of Magnesium Alloys to Corrosion Fatigue for Biodegradable Implant Applications: Current Status and Challenges

**DOI:** 10.3390/ma10111316

**Published:** 2017-11-16

**Authors:** R. K. Singh Raman, Shervin Eslami Harandi

**Affiliations:** 1 Department of Mechanical & Aerospace Engineering, Monash University, Melbourne, VIC 3800, Australia; shervin.harandi@monash.edu; 2 Department of Chemical Engineering, Monash University, Melbourne, VIC 3800, Australia

**Keywords:** biodegradable implants, magnesium alloys, corrosion fatigue

## Abstract

Magnesium (Mg) alloys are attracting increasing interest as the most suitable metallic materials for construction of biodegradable and bio-absorbable temporary implants. However, Mg-alloys can suffer premature and catastrophic fracture under the synergy of cyclic loading and corrosion (i.e., corrosion fatigue (CF)). Though Mg alloys are reported to be susceptible to CF also in the corrosive human body fluid, there are very limited studies on this topic. Furthermore, the in vitro test parameters employed in these investigations have not properly simulated the actual conditions in the human body. This article presents an overview of the findings of available studies on the CF of Mg alloys in pseudo-physiological solutions and the employed testing procedures, as well as identifying the knowledge gap.

## 1. Introduction

**Figure 1:**
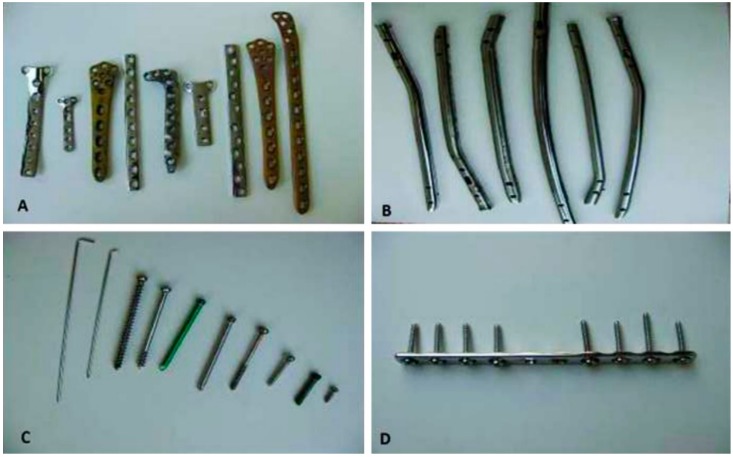
Different types of post-service metallic implants: (**A**) plates of stainless steel and titanium; (**B**) intramedullary nails of stainless steel; (**C**) screws and pins of stainless steel and titanium; (**D**) a plate with stainless steel screws [4].

Deterioration or fracture of bones is among the foremost health concerns, and fixing fracture/deterioration may require the use of permanent and or temporary implants. The temporary implants such as screws, nails, plates and pins are generally removed by second surgery after the bone tissues have healed (Figure 1). Such second surgical procedures currently place a considerable extra burden on both patient and surgical personnel, and avoiding this procedure is obviously a socially and commercially highly attractive proposition. Therefore, employing metallic materials that degrade in a physiological environment without harmful effects has become a matter of increasing research interest for temporary implant applications. The core requirements for a metal/alloy to be suitable as biodegradable implants are their ability to provide adequate support for regenerating bones over the healing period, and subsequently degrade away steadily, completely and without any harmful side effect [1,2,3].

Magnesium (Mg) possesses such specific attributes. Not only is Mg completely biocompatible with human physiology, its mechanical compatibility with human bones is much superior to those of the traditional metallic implant materials, viz, titanium alloys and stainless steels [5,6]. The degradation products of Mg are non-toxic to human physiology, but only when Mg is allowed to degrade slowly in human body fluid (HBF). In spite of the above-mentioned advantages, there are some limitations in the case of Mg as a temporary implant. Mg and its alloys corrode at such rapid rates in HBF that render their mechanical integrity unacceptable, besides generating intolerable amounts of hydrogen. An immoderate amount of hydrogen gas generates an undesirable condition, producing subcutaneous gas bubbles that can separate layers of tissues and block the bloodstream. A further challenge in the application of Mg alloys as biodegradable implants is the alloy design. For implant applications, it is important to identify Mg alloys with alloying elements that confer strength and corrosion resistance in HBF but most importantly, without being toxic to the human body. However, metallic ions released from certain Mg alloys are not always biocompatible as they can disrupt tissue/bone healing. What is more relevant in the context of this article is that the synergistic effect of mechanical loading and the corrosive HBF can cause sudden cracking/fracture of the implants due to the phenomena of stress corrosion cracking (SCC) and corrosion fatigue (CF). SCC susceptibility of Mg alloys in pseudo-physiological solutions has received significant attention, whereas there have been very limited studies on the CF of suitable Mg alloys in HBF. Although these limited studies have confirmed Mg alloys to be generally susceptible to CF, the tests in these studies have been carried out under conditions that do not actually represent the conditions experienced by the temporary implants in human body. The main aims of this article are to present a brief review of the fatigue testing procedures and the findings of the reported studies on the CF of Mg alloys for temporary implant applications. The article also highlights the knowledge gap and the nature of the required investigations.

## 2. Corrosion Fatigue (CF) of Mg Alloys

**Figure 2:**
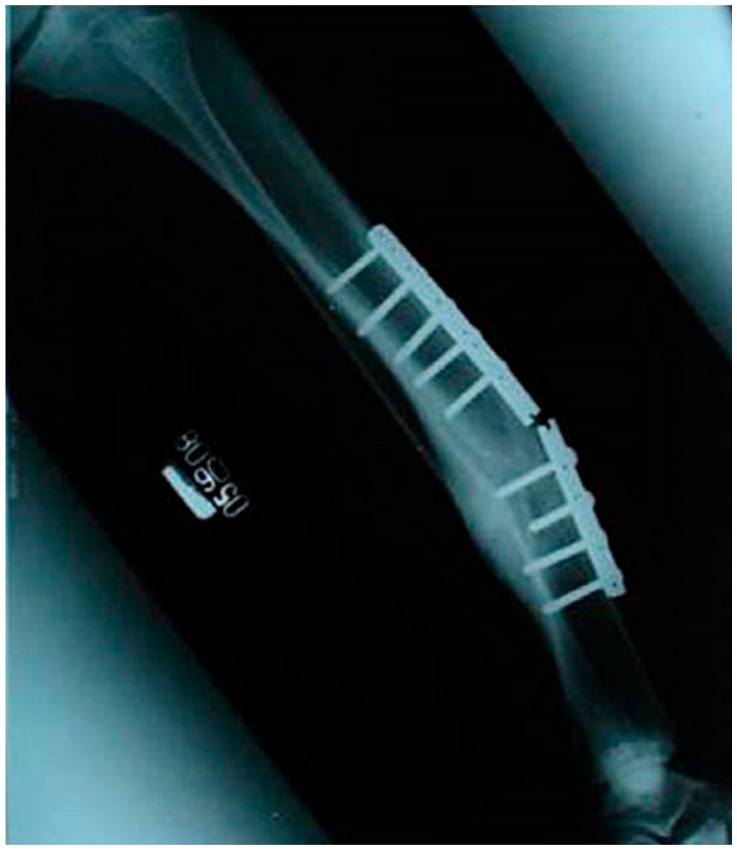
Radiographic image of a failed stainless steel implant due to fatigue [17].

Corrosion fatigue (CF) is the cracking/fracture due to the simultaneous action of cyclic loading and corrosion. CF is reported to cause premature and catastrophic failures of traditional metallic implant materials such as stainless steels (Figure 2) [7,8,9]. Therefore, it is necessary to establish CF characteristics of biodegradable Mg alloys before the alloys are actually employed.

Cracking of Mg alloys under the combined influence of mechanical loading and corrosion is profoundly influenced by the alloying contents [10,11,12,13,14,15,16]. The most common Mg alloys possess Al and Zn as the primary alloying elements (i.e., AZ series alloys), because Al profoundly improves corrosion resistance, whereas Zn effectuates solid solution strengthening of the alloys. Other common alloying elements or impurities of Mg alloys include Fe, Ca and rare earths (REs). Besides their influences in strengthening or corrosion resistance, the primary requirements of the choice of the alloying elements for Mg alloys for bioimplant applications are the possibility and extent of toxicity due to the given element. Al is widely reported to cause neurological disorders. However, Al-containing alloys are the most investigated Mg alloys. Accordingly, Mg alloys for bioimplant applications can be categorised on the basis whether it contained Al.

### 2.1. CF of Al-Containing Mg Alloys

Though Al is widely reported to cause neurological disorders such as Alzheimer’s disease and dementia [18,19], the *in-vivo* and *in-vitro* cytocompatibility tests have suggested the Al release from the Al-containing Mg alloys investigated in a few studies to be within the tolerance limits for implant applications [20,21]. Even though Al-containing alloys (AZ series) are the most investigated Mg-alloys, there are very few studies on the CF of such alloys in HBF. Gu et al. [22] and Jafari et al. [23] investigated the CF of die-cast AZ91D alloy (Table 1) in a simulated body fluid (SBF) at 37 °C, and found that the alloy tested in SBF suffered a significant loss in the mechanical properties under cyclic loading. These deteriorations in mechanical properties could be the result of the susceptibility of the alloy to the CF in SBF. Table 2 describes the fatigue limits, and fatigue testing procedures and test conditions employed in these studies. Fractography of the failed specimens suggested the cracks to have initiated due to localized corrosion (pitting) in SBF, whereas during tests in air, the cracks initiated at MgO inclusions. Since the environment-assisted cracking (corrosion fatigue and stress corrosion cracking) is profoundly influenced by electrochemical condition at the crack-tip, Jafari et al. [10] have also investigated the effect of imposed continuous cathodic and anodic charging conditions on corrosion fatigue resistance of the alloy. These experiments indicated a significant difference in CF strength under both the electrochemical conditions when tests were performed at stresses lower than the fatigue limit in air (57 MPa). Accordingly, hydrogen embrittlement was inferred to have played a dominant role in cracking of the alloy in m-SBF, as a result of the stress-assisted diffusion of hydrogen at high-stress intensity area ahead of the crack-tip [23,24,25].

Although the studies by Gu et al. [22] and Jafari et al. [23] have confirmed the AZ alloys to be generally susceptible to CF, there is a critical knowledge gap in understanding the CF of Mg alloys from a comprehensive biomedical perspective. The two studies [22,23] were carried out under simple axial loading (tension–compression) whereas the implants in actual body environment often experience more complex loading conditions. For instance, a femoral implant undergoes cyclic bending loading even during normal activities such as walking or running [27]. Therefore, the evaluation of resistance of Mg alloys as the femoral midshaft implant necessitates evaluation of CF under bending. Accordingly, in a recent study [26], bending was the employed loading mode when the mechano-chemical conditions were designed for CF tests for appropriately simulating the actual human body environment. The testing procedures and test conditions employed in this study are shown in Table 2. Other measures employed in this study for simulation of the mechano-chemical conditions as well as for proper comparison of the *in-vitro* CF results out of these tests vis-à-vis those of *in-vivo* testing [28] are: (a) the dimensions of the cylindrical test specimens (diameter: 2 mm, length: 50 mm) were similar to the implants that can be used for *in-vivo* tests, (b) maximum stresses (17 N) as well the cycling frequency (1 Hz) were both low and (c) pH of the test solution was controlled by purging CO_2_ (as opposed to the pH control by using chemicals such as HEPES or Tris, as shown in Table 2). This study indicated the alloy to have a fatigue limit of 142 MPa in air, and a corrosion fatigue resistance of 101 MPa at ~25 × 10^3^ cycles in Hanks’ solution and 10^4^ cycles in the plain Hanks’ solution that also contained bovine serum albumin (BSA). BSA is the most abundant protein in human blood plasma.

Another interesting finding of this recent study employing more appropriate mechano-chemical conditions [26] was the beneficial effect of BSA addition in retarding the CF crack propagation at the early stages when the constant stress levels were lower than the fatigue limit in air (142 MPa). However, it must be emphasised that this is the first study on the CF of a Mg alloy under more appropriately simulated mechano-chemical conditions, and there is a need for more comprehensive studies on this topic, particularly those that are designed taking into account the different loading conditions of the human body as well as other physiological constituents of the human body fluid, such as amino acids, glucose, fibrinogen, etc.

### 2.2. Corrosion Fatigue of Al-Free Mg Alloys

Even though there is no compelling evidence to support the notion that the Al release from the Al-containing Mg-alloys (AZ series alloys) could be at the toxic levels for human physiology, the research and technology community is at a cross-road on this point [29]. However, in actual practice, the Al-containing Mg-alloys are generally not considered for implant applications. Taking into account the non-toxic nature of various metals, Ca, Zn and rare earths (REs) are the common elements that have been used for alloying of the Al-free Mg-alloys for bioimplant applications.

Calcium (Ca) is a major component in human bones. Ca-containing Mg-alloys quickly develop a surface layer of hydroxy apatite, improving the compatibility of the alloy with the human body [30]. Ca refines Mg-alloy grain size and improves both their mechanical properties and corrosion resistance [1,31]. However, at ≥1 wt %, Ca forms Mg_2_Ca precipitates along alloy grain boundaries that cause embrittlement and impair the mechanical properties [30]. Human physiology requires ~15 mg of Zn each day [32]. Zn causes solid solution strengthening of Mg, but, at ≥6.2 wt %, Zn starts to form Mg–Zn precipitates [33], again causing embrittlement. Mg–Ca, Mg–Zn and Mg–Zn–Ca alloys [30,34,35,36] have been investigated for bio-implant applications and these alloys were found to meet non-toxicity requirements (as per the cyto-toxicity tests). Hydrogen generation due to corrosion of Mg-alloys in human body fluid is another traditional problem in using Mg alloys. However, the development of very recent Mg–Zn–Ca alloys [35] seems to have considerably addressed this problem.

The addition of rare earths (REs) improves the creep resistance as well as corrosion resistance of Mg alloys. REs are generally believed to be non-toxic [37], but there are also contrary reports. REs readily form very fine and stable intermetallic precipitates, and hence, are very effective in strengthening, even when present in small quantities. However, these intermetallics when present in sufficient size and quantity can cause localized corrosion and embrittlement. However, a few REs (e.g., Y, Gd, Yb, Tb, Dy, Ho, Er, Th) have much greater solubilities in Mg (than other REs) [37], thereby providing a window of opportunity to select one or more REs in their tolerable quantities for alloying with Mg, that will confer the required corrosion resistance while minimizing intermetallic formation. A recently developed Mg–Zn–Ca–Y alloy [35,38] was found to possess the strength and corrosion resistance for the required duration of likely use as temporary implants.

Very few investigations have reported on the fatigue and CF of Al-free Mg alloys in HBF/SBF [39,40]. The testing procedures for various alloys, other experimental parameters and main findings are summarized in Table 3. These alloys showed fatigue limits in SBF. With the exception of Mg–1Zn–0.3Ca alloy, fractography suggested the fatigue crack in all Al-free Mg alloys to have nucleated from the microstructural defects when tested in air. In the case of the Mg–1Zn–0.3Ca alloy, twin boundaries were reported as the crack nucleation site. When the Al-free Mg alloys were tested in SBF, the crack initiated from surface corrosion pits.

The studies listed in Table 3 have provided important mechanistic insight into the CF of Al-free Mg alloys in a physiological environment. However, there is a need for investigation of the CF of Mg alloys under more appropriately mechano-chemical conditions, similar to those reported previously [26] (and briefly described in Section 3). Such investigations need to be more comprehensive, particularly taking into account the different loading conditions of the human body as well as other physiological constituents of the human body fluid, such as BSA, amino acids, glucose, fibrinogen etc.

The Al-free Mg-alloys employed in the studies described in the preceding paragraphs have all possessed a crystalline microstructure. However, a critical study showed that when the structure of such alloys is amorphous, their corrosion rate is drastically retarded and hydrogen generation that is concurrent with Mg corrosion is suppressed to a level that is clinically not observable [34]. Among the Mg-based glass systems, Mg–Zn–Ca amorphous alloys have been shown to possess the best biocompatibility both in vitro and in vivo [34,41,42,43]. Gu et al. [41] reported that Mg–Zn–Ca metallic glasses exhibit three times the strength of pure Mg. This beneficial effect of the amorphous microstructure has been attributed to the homogenous single solid-solution phase [42,44], i.e., the absence of second phase in the alloy system [41,45,46,47] in corrosion resistance as well as the mechanical properties. Although there is significant reported literature on the corrosion behaviour and mechanical properties of amorphous Mg–Zn–Ca for biodegradable implant applications, the CF resistance of these amorphous alloys has received very little attention in the only study. Li et al. [48] have recently evaluated the CF of an amorphous Mg_66_Zn_30_Ca_3_Sr_1_ in a simulated physiological environment. Table 4 compares the fatigue resistance of this amorphous alloy with another Al-free alloy that is crystalline. The amorphous Mg_66_Zn_30_Ca_3_Sr_1_ alloy showed much superior fatigue and corrosion fatigue resistance than the crystalline alloy [22,48]. 

## 3. Recommendation to Further Work

The reported studies have attempted to investigate the corrosion fatigue (CF) of Mg alloys under conditions closer to the actual body environment. However, there are other critical aspects that need further exploration and investigation as outlined below:

- Although the effect of BSA as the most abundant protein in human blood plasma was investigated on the CF of Mg alloys by the authors [26], body plasma consists of a large amount of other organic compounds such as amino acids, glucose, fibrinogen [6,27]. Therefore, it is of utmost importance to pursue studies on the possible role of the combination of all organic elements on corrosion and corrosion-assisted cracking (including CF) of Mg alloys.
- As described earlier, body implants are subjected to acute and complex loading during service conditions. However, while running or jumping, significantly different loading characteristics are experienced by the medical implants. Therefore, it is necessary to carry out tests under specific loading patterns for a given temporary implant in the actual human body environment.

## 4. Conclusions

Because of the importance of the resistance of bioimplant devices to degradation and fracture in the actual service, recent investigations on corrosion-assisted cracking of magnesium (Mg) alloys as potential bioabsorbable implant materials have become the focus of interest. The reported investigations in simulated body fluid (SBF) have shown Mg alloys to be susceptible to both stress corrosion cracking (SCC) and corrosion fatigue (CF), on the basis of both mechanical data and fractographic evidence, and pitting to be the common crack initiator. However, in comparison with SCC, studies on CF of Mg alloys in a physiological environment have received very little attention. The reported investigations on CF behaviour of Al-containing and Al-free Mg alloys have been carried out under conditions that are considerably removed from the conditions actually experienced by implants in the human body environment. This article identifies the critical knowledge gaps, such as the understanding of the role of addition of organic components to SBF and the mechanical testing parameters in CF of Mg alloys. In addition, in order for in vitro studies to be more meaningful and valuable to clinical investigations, this review provides a pathway for appropriate investigations for developing Mg alloys with the required resistance to cracking or fracture in the actual human physiological environment. This review identifies more accurate testing parameters (namely, test alloys, frequency and mode of loading, and chemistry of the test solutions that appropriately simulate the actual human body conditions).

## Figures and Tables

**Table 1:** Chemical composition of AZ91D and WE43 alloys (wt %).

Element	Mg	Al	Zn	Mn	Cu	Fe	Ni	Si	Be	Y	RE	Zr	Ref.
AZ91D	Bal	8.89	0.78	0.20	0.002	0.002	<0.001	<0.01	<0.001	-	-	-	[23]
	89.59	9.21	0.80	0.34	-	-	-	0.06	-	-	-	-	[22]
WE43	91.35	-	0.20	0.13	-	-	-	-	-	4.16	3.80	0.36	[22]

**Table 2:** Comparison of fatigue limits and experimental set-ups employed for evaluation of the fatigue life of a common Al-containing Mg alloy in different pseudo-physiological solutions.

Alloy	Fatigue Limit (MPa)		Number of Cycles (N)		The Testing Procedure and Test Conditions						
	Air	Medium	Air	Medium	Medium	pH Controller	Temperature (°C)	Loading	Stress Ratio	Frequency (Hz)	Ref.
AZ91D	50	20	10^7^	10^6^	SBF	Tris	37	Tension–compression	−1	10	[22]
AZ91D	57	17	10^7^	5 × 10^5^	m-SBF	HEPES	37	Tension–compression	−1	5	[23]
AZ91D	142	101	10^6^	~25 × 10^3^ (In Hanks’ solution)	Hanks’ solution + BSA	Purging CO_2_	37	Three-point bending	0.1	1	[26]
				10^4^ (In Hanks’ solution + BSA)							

SBF = simulated body fluid; m-SBF = modified simulated body fluid; BSA = bovine serum albumin; HEPES = hydroxyethyl-piperazine ethanesulafonic acid.

**Table 3:** Comparison of fatigue limits and experimental set-ups employed to evaluate the fatigue life of Al-free Mg alloys in SBF.

Alloy	Fatigue Limit (MPa)		Number of Cycles (N)		The Testing Procedure and Test Conditions						
	Air	Medium	Air	Medium	Medium	pH Controller	Temperature (°C)	Loading	Stress Ratio	Frequency (Hz)	Ref.
WE43 *	110	40	10^7^	10^7^	SBF	Tris	37	Tension–compression	−1	10	[22]
Mg-1Ca	~90	70	4 × 10^6^	4 × 10^6^	SBF	Tris	37	Tension–compression	−1	10	[39]
Mg–2Zn–0.2Ca	~90	68	4 × 10^6^	4 × 10^6^	SBF	Tris	37	Tension–compression	−1	10	[39]
Mg–1Zn–0.3Ca	~106 (E325)	~60 (E325)	10^7^	5 × 10^6^	m-SBF	HEPES	37	Tension–compression	−1	10	[40]
	~81 (E400)	~60 (E400)									

E325 = Mg–1Zn–0.3Ca alloy processed at extrusion temperature of 325 °C; E400 = Mg–1Zn–0.3Ca alloy processed at extrusion temperature of 400 °C; SBF = simulated body fluid; m-SBF = modified simulated body fluid; BSA = bovine serum albumin; HEPES = hydroxyethyl-piperazine ethanesulafonic acid; * = the chemical composition of this alloy is shown in Table 1.

**Table 4:** Comparison of the fatigue resistance of amorphous Mg–Zn–Ca–Sr alloy and crystalline WE43 alloy.

Alloy	Fatigue Strength (MPa)		The Testing Procedure Test Conditions					
	Air	Medium	Number of Cycles (N)	Medium	Loading	Stress Ratio	Frequency (Hz)	Ref.
Mg–Zn–Ca–Sr (Amorphous)	370	150	10^7^	PBS	Compression–compression	0.1	10	[48]
WE43	110	40	10^7^	SBF	Tension–compression	−1	10	[22]

PBS = phosphate-buffered saline; SBF = simulated body fluid.
